# Smell, an Underrated Early Biomarker for Brain Aging

**DOI:** 10.3389/fnins.2020.00792

**Published:** 2020-08-25

**Authors:** Emanuele Brai, Thomas Hummel, Lavinia Alberi

**Affiliations:** ^1^Center for Brain and Disease Research, Flanders Institute for Biotechnology (VIB) – Catholic University (KU) Leuven, Leuven, Belgium; ^2^Smell and Taste Clinic, Department of Otorhinolaryngology, University Hospital Dresden, Dresden, Germany; ^3^Swiss Integrative Centre for Human Health (SICHH), Fribourg, Switzerland; ^4^Department of Oncology, Microbiology and Immunology, Faculty of Science and Medicine, University of Fribourg, Fribourg, Switzerland

**Keywords:** smell, Alzheimer’s disease, vision, hearing, biomarkers brain alterations

## Abstract

Olfaction is in addition to touch the most ancient of our senses, developing already in the womb it decays progressively from 65 years of age with a more pronounced impairment associated with dementia. Despite its clinical relevance and testing accessibility, smell remains an overlooked biomarker, which is rarely used by neurologists in the early screening phase. In this perspective article, we outline the reasons underlying the lack of awareness for this sense. In an attempt to put olfaction forward as an early biomarker for pathological brain aging, we draw a comparison with vision and hearing, regarded as more relevant for general health. This perspective article wants to encourage further studies aimed at understanding the mechanisms responsible for the early smell dysfunction in individuals a decade or more before the onset of cognitive symptoms.

## Smell – An Invisible but Vital Sense

Olfaction, from an evolutionary aspect, is the oldest of our senses. Across different species, it modulates the interactions between an organism and the surrounding environment even before birth (Brai and Alberi, 2018). From early postnatal life into adulthood, the sense of smell regulates many of our behaviors, from nutrition to social interaction (Schaal et al., 2020). For example, human neonates are attracted by a pad impregnated with the mother’s breast odor (promoting locomotor activity) compared to an empty control pad, supporting an odor memory developed already during the fetal stage (Han and Hummel, 2019). Nevertheless, the majority of the studies on chemo-sensation have been developed in rodents, with a less rich literature in humans (Zhou et al., 2019). The incomplete understanding of human olfaction may relate to the complexity of studying the multiple olfactory centers distributed in several brain regions comprising the cortical and the subcortical pathways, e.g., olfactory bulb, piriform and entorhinal cortex, amygdala, orbitofrontal cortex and hypothalamus (Shepherd, 2004; Atanasova et al., 2008; Fagundo et al., 2015; Hummel et al., 2017). This anatomical heterogeneity implies an extensive connection among the olfactory sensory areas which constitute a complex network essential to associate the olfactory stimulus with other cerebral regions, such as those involved in the processing of memories and emotions and multisensory integration with other senses (Shepherd, 2004; McGann, 2017).

Another challenge facing smell research in humans relates to its minor clinical implication as compared to impairment of vision and hearing: the occurrence of blindness or deafness produces a massive personal and social deficit which severely disrupts someone’s life. In line with these observations, the different attention paid to these three senses has been also described by Murphy et al. (2002) who reported that older adults in the US received assistance for vision and hearing deficits, whereas no testing for olfactory dysfunction was performed. While vision and hearing have been treated as primary senses for general health, olfaction is gaining increasing importance in clinical settings since its impairment represents an overarching non-invasive biomarker in predicting dementia during aging (MacDonald et al., 2018). With the frequent decline in smell acuity, mostly attributed to the reduced turnover of the olfactory neuroepithelium with aging (Doty et al., 2011), the early and pronounced olfactory deficit described in different neurodegenerative diseases, ranging from Alzheimer’s to Parkinson’s and Huntington’s diseases remains yet poorly understood.

In daily life, smell is an “invisible” sense influencing much of our daily existence from social interactions to our most ancestral memories and healthy aging. Without the sense of smell, there is no flavor perception during eating and drinking, so that everything tastes bland (Croy et al., 2014). Without a functioning sense of smell, many people develop mood and anxiety disorders. This body of evidence can be partially explained from an anatomical perspective since several brain regions, such as the limbic system and the orbitofrontal cortex, are involved in olfactory processing and also in the pathophysiology of these psychiatric disturbances (Atanasova et al., 2008; Stevenson, 2010; Croy et al., 2014; Takahashi et al., 2015; Kohli et al., 2017; Kamath et al., 2018; Rochet et al., 2018).

For instance, Croy and colleagues have shown that depressed patients display a decreased olfactory acuity. In particular, a decrement in odor discrimination and activation of the olfactory areas was observed in these individuals compared to healthy subjects. However, upon anti-depressive intervention, the olfactory parameters were comparable in the two groups (Croy et al., 2014). Additional data supporting the interconnection between olfaction and depression report that a dysfunction in odor identification can also be experienced by patients affected by this disorder (Kamath et al., 2018).

Among the studies suggesting a potential link between anxiety and olfactory impairment, Takahashi and coworkers explored whether anxiety traits could influence olfactory tasks in healthy subjects. Specifically, the authors observed that individuals suffering from states of anxiety presented remarkable odor detection and identification deficits (Takahashi et al., 2015).

Anxiety and mood disorders may impinge the cognitive function in older adults as indicated by neuroimaging data, showing that this condition may be possibly considered as an early marker or an independent risk component for Alzheimer’s disease (AD) (Becker et al., 2018). Furthermore, subjects affected by mild cognitive impairment or dementia show a higher probability to develop anxiety (Lyketsos et al., 2002). Therefore, accounting for olfactory impairment and co-symptomatic depression may represent a multifactorial risk factor for the development of dementia with aging.

Altogether these observations suggest that understanding more deeply how olfactory processing modulates odor perception across the lifespan will add considerable value for our well-being and health status along the age continuum.

## Olfaction, Vision, and Hearing as Non-Invasive Biomarkers in Dementia

**FIGURE 1:**
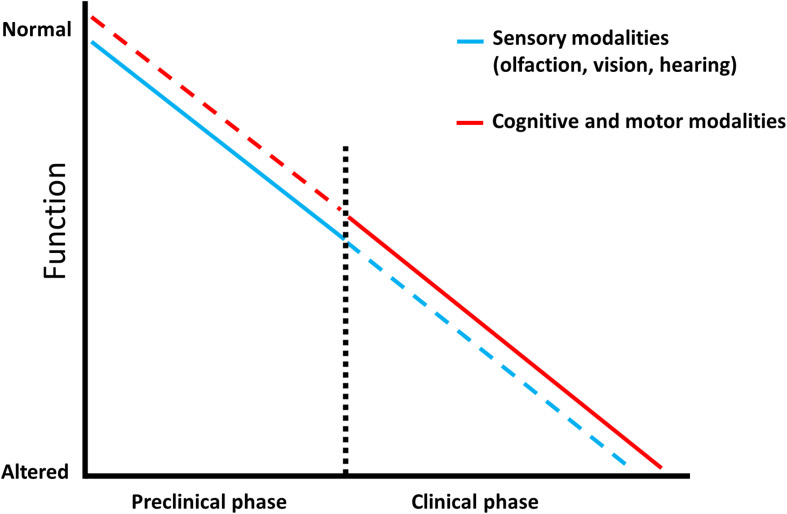
Predictive factors characterizing dementia. Schematic representation showing that sensory modalities are affected from the prodromal phase of Alzheimer’s disease and represent an early prognostic tool (blue line) which is less efficient in the late stage of the pathology, as sensory testing can be influenced by additional impairments (blue dotted line). Complex cognitive and motor abilities display a low predictive power in the preclinical phase since they are mainly intact (red dotted line), however, they have an increased diagnostic value in the clinical stage where they progressively worsen (red line).

In an effort to halt the progressive spread of Alzheimer’s disease worldwide, more studies in the past decade have focused on the senses, vision, hearing, and smell, as sources of stable non-invasive biomarkers (MacDonald et al., 2018; Murphy, 2019; Lin, 2020), which may enable a preventive treatment during its preclinical phase (Figure 1). In this section we summarize some of the advances in sensory biomarkers offering a predictive value for dementia conversion/progression.

### Olfactory Biomarkers

Olfactory performance reaches its peak at the age of 40 years (Buschhüter et al., 2008) and then progressively declines with aging (Eibenstein et al., 2005; Doty and Kamath, 2014; Palmquist et al., 2020; Parvand and Rankin, 2020; Van Regemorter et al., 2020). In addition, in different neurological pathologies, like Parkinson’s and Alzheimer’s disease, dysosmia is exacerbated compared to the physiological decrease and occurs prior to motor and cognitive disabilities (Doty, 2012; Masala et al., 2018; Bathini et al., 2019; Cecchini et al., 2019; Dintica et al., 2019; Murphy, 2019). Many studies sustain that the alteration of the olfactory system may be used as an early predictor for detecting AD because several brain olfactory regions are impaired in the asymptomatic phase of this disorder due to the deposition of pathological hallmarks (Braak and Braak, 1991). Consequently, monitoring the olfactory sensitivity through different tests offers a supplementary approach which, together with other medical evaluations, can help to forecast the risk of developing memory decline, favoring the implementation of prevention strategies and promotion of clinical trials development (Table 1).

**TABLE 1:** Sensory biomarkers and their predictive power to diagnose Alzheimer-related dementia at early stages.

Non-invasive biomarker	Predictive power	Type of test/deficit	References
**Olfaction**	+++	Odor identification	Wilson et al., 2007b; Lötsch et al., 2008; Hummel et al., 2010; Rahayel et al., 2012; Doty and Kamath, 2014; Doty, 2015; Quarmley et al., 2017; MacDonald et al., 2018
		Odor discrimination	Lötsch et al., 2008; Rahayel et al., 2012; Sohrabi et al., 2012; Doty and Kamath, 2014; Quarmley et al., 2017
		Threshold	Lötsch et al., 2008; Rahayel et al., 2012; Doty and Kamath, 2014; Doty, 2015; Quarmley et al., 2017
		Swabs and biopses	Brozzetti et al., 2020

**Vision**	++	Retinal	Pike et al., 2007; Rowe et al., 2007; Javaid et al., 2016; Lim et al., 2016; Colligris et al., 2018; O’Bryhim et al., 2018; Alber et al., 2020
		Non-retinal	Javaid et al., 2016; Lim et al., 2016; Colligris et al., 2018; Dehghani et al., 2018
		Visual acuity	Harding et al., 1981; Coben et al., 1983; Schlotterer et al., 1984; Cogan, 1985; MacDonald et al., 2018
		Tear fluid	Kalló et al., 2016

**Hearing**	++	Central auditory testing	Gates et al., 2008; Lin, 2020
		Pure tone threshold	Gates et al., 2008; Lin, 2011, 2020; Lin et al., 2011a, b; MacDonald et al., 2018
		Word recognition	Gates et al., 2008; Merten et al., 2020

There is a battery of tests which can be adopted to gauge olfactory loss (Doty and Kamath, 2014), but the most used in determining early-stage AD include odor identification, discrimination and threshold (Lötsch et al., 2008; Hummel et al., 2010; Doty, 2015).

In particular, the first two olfactory tasks provide a more accurate evaluation in diagnosing prodromal dementia (Rahayel et al., 2012; Sohrabi et al., 2012; Quarmley et al., 2017). This can be explained by the fact that odor discrimination and identification rely, to a greater extent than odor threshold, on higher brain centers, such as the piriform, entorhinal, orbitofrontal cortices and the amygdala (Rahayel et al., 2012; Sohrabi et al., 2012; Quarmley et al., 2017), therefore indicating a more complex processing for their execution. On the contrary, odor threshold performance is mainly associated with peripheral olfactory stimuli as perceived by the olfactory receptors placed in the nasal neuroepithelium (Doty et al., 1994; Atanasova et al., 2008) and is susceptible to changes in the anatomy of the nasal cavity and clogging of the cribriform plate which occurs naturally with aging (Damm et al., 2002; Doty and Kamath, 2014; Masala et al., 2019).

Olfactory function can also be influenced by other factors, e.g., smoking or chronic rhinosinusitis may also trigger olfactory loss and should be taken into consideration (Devanand, 2016). Psychophysical tests of olfactory function are non-invasive, fast, inexpensive, and may be self-administered. Moreover, they can spare some patients from undergoing costly examinations, such as positron emission tomography (PET) scans, especially when administered together with other analysis (Devanand, 2016). In order to obtain reliable results, an important aspect which is taken into account when performing odor identification tasks is that people from different regions are familiar with distinct odors related to their cultural and traditional background (Konstantinidis et al., 2008; Neumann et al., 2012; Tekeli et al., 2013; Cavazzana et al., 2017). In addition to olfactory tests, nasal biopsies and swabs represent a supplementary procedure which can be adopted to evaluate the integrity of the olfactory system and its possible impairment in neurodegenerative diseases (Brozzetti et al., 2020). This multivariate approach on studying the olfactory system may provide complementary results useful for the diagnosis of neurological pathologies during their preclinical phase, therefore further elucidating whether a person is “silently” developing a pathology.

In conclusion, fully characterizing olfaction in its diverse aspects, as done with the other senses, will provide an added value in understanding the development of cognitive deficits and their prevention, offering new horizons for therapeutic applications during the preclinical phase of AD and possibly tackling its progression before it becomes irreversible.

### Visual Biomarkers

Additionally to olfactory decline, AD patients can experience visual disturbances before displaying memory impairment (Harding et al., 1981; Coben et al., 1983; Schlotterer et al., 1984). Poor vision, spatial disorientation, and visual agnosia were characterized in patients with presumable AD (Cogan, 1985). Therefore, detecting these deficits in the asymptomatic stage of the pathology might play an important role in its early diagnosis (Javaid et al., 2016; Lim et al., 2016; Colligris et al., 2018; Alber et al., 2020). There is a number of retinal and non-retinal biomarkers which may be used to predict the risk of dementia in its prodromal phase (Javaid et al., 2016; Lim et al., 2016; Colligris et al., 2018; Dehghani et al., 2018; Alber et al., 2020; Table 1). For instance, some reports show that eye tests conducted using PET and optical coherence tomographic angiography could detect retinal microvascular abnormalities in cognitively normal subjects with preclinical AD (O’Bryhim et al., 2018). Furthermore, PET analysis revealed that intraretinal aggregation of β-amyloid and Tau occurs before the onset of AD clinical profile (Pike et al., 2007; Rowe et al., 2007). In particular, the structural variation observed in the eye components along with the accumulation of amyloid deposits cause visual deficits in the early stages of AD, and such variations could also alter the composition and the production of tears. Notably, it has been shown that the protein level in this ocular fluid is increased in AD subjects compared to that in healthy controls (Kalló et al., 2016). This data is in line with a previous study describing the use of tears to detect other neurodegenerative pathologies, like glaucoma (Tezel, 2013). While the detection of structural and functional changes through retinal scans may be informative of early neurodegenerative processes, it requires medical assistance and will be less accessible and compliant than olfactory testing to aging adults. On the other hand, point-of-care tear biomarker diagnostics may have a wider acceptance and usability across the population, but studies in this area remain few to date and there is so far no approved or marketed product.

### Auditory Biomarkers

During aging the prevalence of hearing reduction becomes predominant and it is estimated that about 9–14% of people aged >65 years show auditory processing disorders (Rosenhall, 2015). With age-dependent hearing damage communication is affected, contributing to social isolation and loneliness which may alter the integrity of cognitive processes (Bennett et al., 2006; Wilson et al., 2007a). In addition, increasing studies suggest the association between hearing loss and cognitive deficit and dementia-related disorders (Gates et al., 2008; Lin, 2011; Lin et al., 2011a, 2014). Furthermore, such deficits appear to be stronger in concurrent multi-sensory impairments, like in auditory and vision domains (Yamada et al., 2016). Auditory performance can be tested through several measures, such as pure tone threshold, word recognition and central auditory testing (Gates et al., 2008; Lin, 2011, 2020; Lin et al., 2011a, b; Merten et al., 2020) (Table 1). Overall, hearing loss alters the input and therefore the information from external stimuli, leading to reduction in general awareness of the person with respect to time and space. Taken altogether, these observations sustain a potential involvement of hearing loss in dementia and to the late AD pathobiology. However, further studies need to target the key mechanisms underlying the link between auditory dysfunction and dementia/AD condition and therefore promote hearing monitoring as a reliable early predictor of cognitive decline.

## Conclusion

In the challenge for finding relevant diagnostic markers to target dementia in its early stage, sensory modalities are offering themselves as promising non-invasive predictors. Different studies provide increasing evidence that monitoring olfaction acuity and, to some extent, vision and hearing could contribute to the early detection of neuronal changes linked to mental decline, therefore promoting a more immediate intervention long before the onset of the cognitive symptoms (Table 1).

As shown by several studies, a reduced olfactory activity is a valuable predictor for cognitive decline in non-demented older adults (Wilson et al., 2007b; Sohrabi et al., 2012). The sequential association between olfactory dysfunction and cognitive deficit is not completely resolved. Nevertheless, the spreading rostral to caudal tauopathy in the limbic brain areas may influence olfactory transmission first and cognitive processing later (Doty and Kamath, 2014; Dintica et al., 2019). These observations further support the association between the sense of smell and cognition and promote olfactory testing to identify those subjects with a higher risk of developing dementia.

Since visual symptoms are experienced by many AD patients, increasing studies are investigating whether this sensory system may represent another potential source of early biomarkers to diagnose cognitive decline and dementia. Among the features corroborating the pathophysiological link between visual/ocular impairments and cognitive dysfunction, it can be acknowledged that the eye and the central nervous system have some commonalities, such as the same embryologic origin and the presence of physiological barriers (Alber et al., 2020). In addition, advanced imaging techniques revealed the accumulation of AD hallmarks in visual structures during the prodromal stage of dementia (Lim et al., 2016; Colligris et al., 2018; Alber et al., 2020).

Many research projects foster the use of hearing assessment as a supplemental diagnostic measure to target cognitive decline and dementia during their preclinical stage. For instance, Lin et al. (2011a, b) explored whether hearing impairment could be mirrored by decreased cognitive tests scoring. Interestingly, they observed that hearing decrement was related with lower cognitive scores. Further evidence proposing this interplay have been correlated with cerebral atrophy in regions implicated in speech processing, common degenerative mechanisms, social isolation and altered communication (Lin et al., 2011a, 2014; Lin and Albert, 2014).

Overall, if the use of sensory functions would be considered as a reliable tool to evaluate the risk of developing dementia, this would enrich the array of multi-step screening protocols that can be adopted in clinical practice. As with other chronic pathologies, such as diabetes and hypertension, where a medical evaluation is regularly recommended in many countries, the monitoring of olfactory performance during aging through simple smell testing could inform about a possible neuropathological risk. A strong informative campaign about the potential benefits which could be obtained by administering olfactory tests might lead to a broader consensus in providing a first screening phase in classifying individuals with a higher risk of developing cognitive impairment and then fostering the development of novel clinical trials. Obviously, supplementary examinations like cognitive tests and brain imaging techniques would be required to confirm this evidence.

In conclusion, we see olfactory testing as a potentially first-line non-invasive diagnostic measure with great compliance and flexibility that may inform the patient and the family and aid in planning healthcare interventions. This primary monitoring would be of substantial value for clinical studies where the selection and the recruitment of subjects in prodromal stages remain challenging. Furthermore, a first diagnostic screening based on olfactory testing will allow the swift implementation of preventive approaches aimed at improving cognition and brain health. In the future, we see the integration of analog tests with IT interfaces expanding the capabilities of olfactory monitoring as part of the personalized health program aimed at improving the quality of life for a long term.

## Data Availability Statement

All datasets generated for this study are included in the article/supplementary material, further inquiries can be directed to the corresponding author.

## Author Contributions

EB wrote the manuscript. TH reviewed and gave the feedback. LA advised and wrote part of the text. All authors contributed to the article and approved the submitted version.

## Conflict of Interest

The authors declare that the research was conducted in the absence of any commercial or financial relationships that could be construed as a potential conflict of interest.
